# Screening and staging of chronic obstructive pulmonary disease with deep learning based on chest X-ray images and clinical parameters

**DOI:** 10.1186/s12890-024-02945-7

**Published:** 2024-03-26

**Authors:** XiaoLing Zou, Yong Ren, HaiLing Yang, ManMan Zou, Ping Meng, LiYi Zhang, MingJuan Gong, WenWen Ding, LanQing Han, TianTuo Zhang

**Affiliations:** 1 Department of Pulmonary and Critical Care Medicine, The Third Affiliated Hospital of Sun Yat-sen University, Institute of Respiratory Diseases of Sun Yat-Sen University, 600 Tianhe Road, Guangzhou, 510630 China; 2 Scientific research project department, Guangdong Artificial Intelligence and Digital Economy Laboratory (Guangzhou), Pazhou Lab, Guangzhou, China; 3 Shensi lab, Shenzhen Institute for Advanced Study, UESTC, Shenzhen, China; 4 Department of Pulmonary and Critical Care Medicine, Dongguan People’s Hospital, Dongguan, China; 5 Department of Pulmonary and Critical Care Medicine, the Six Affiliated Hospital of Guangzhou Medical University, Qingyuan People’s Hospital, Qingyuan, China; 6 Department of Internal Medicine, Huazhou Hospital of Traditional Chinese Medicine, Huazhou, China; 7 Center for artificial intelligence in medicine, Research Institute of Tsinghua, Pearl River Delta, Guangzhou, China

**Keywords:** COPD screening, Pulmonary function test, Deep learning models, Chest X-ray, Clinical parameters

## Abstract

**Background:**

Chronic obstructive pulmonary disease (COPD) is underdiagnosed with the current gold standard measure pulmonary function test (PFT). A more sensitive and simple option for early detection and severity evaluation of COPD could benefit practitioners and patients.

**Methods:**

In this multicenter retrospective study, frontal chest X-ray (CXR) images and related clinical information of 1055 participants were collected and processed. Different deep learning algorithms and transfer learning models were trained to classify COPD based on clinical data and CXR images from 666 subjects, and validated in internal test set based on 284 participants. External test including 105 participants was also performed to verify the generalization ability of the learning algorithms in diagnosing COPD. Meanwhile, the model was further used to evaluate disease severity of COPD by predicting different grads.

**Results:**

The Ensemble model showed an AUC of 0.969 in distinguishing COPD by simultaneously extracting fusion features of clinical parameters and CXR images in internal test, better than models that used clinical parameters (AUC = 0.963) or images (AUC = 0.946) only. For the external test set, the AUC slightly declined to 0.934 in predicting COPD based on clinical parameters and CXR images. When applying the Ensemble model to determine disease severity of COPD, the AUC reached 0.894 for three-classification and 0.852 for five-classification respectively.

**Conclusion:**

The present study used DL algorithms to screen COPD and predict disease severity based on CXR imaging and clinical parameters. The models showed good performance and the approach might be an effective case-finding tool with low radiation dose for COPD diagnosis and staging.

**Supplementary Information:**

The online version contains supplementary material available at 10.1186/s12890-024-02945-7.

## Background

Chronic obstructive pulmonary disease (COPD) is a common pulmonary disease characterized by persistent respiratory symptoms and airflow limitation that is due to airway and/or alveolar abnormalities mainly caused by cigarette smoking [1]. Despite many decades of research on the pathogenesis and treatment of COPD, the medical community has failed to decrease its morbidity and mortality to the same degree that has been achieved in other major noncommunicable diseases, An important factor contributing to this slow progress may be that he previous COPD definition showed limitations as the lack of identification of the disorder at its early stages in the absence of flow limitation [2]. The proposed solutions are aimed to encourage novel treatments and translational studies: incorporating into the definition objectivable early computed tomography (CT) scan changes [3]. Epidemiological studies have shown that COPD is the third leading cause of death and accounts for 5% of all deaths worldwide each year [4]. The overall prevalence of COPD in people aged 40 years and older was 12.64% (95% CI 10.75%-14.65%) and 7.38% [5]. About a quarter of adults over 40 years have moderate airflow limitations, yet most of them are unaware of their conditions [6]. Early diagnosis of COPD is critical for early self-management and timely therapy to improve the overall prognosis [7, 8]. However, a considerable proportion of COPD patients are undiagnosed. An estimate of over 40% of COPD patients remain undiagnosed, particularly in developing countries [9, 10], and only 12% of individuals with chronic airflow limitations had a previous spirometry-defined COPD diagnosis during the recent screening of 57,779 participants in China [11].

The conventional diagnosis and staging measure of COPD is pulmonary function test (PFT) according to the Global Initiative for Chronic Obstructive Lung Disease (GOLD) diagnostic criteria [12]. However, spirometry strongly depends on patients’ cooperation and COPD can either be misdiagnosed or missed entirely when using spirometry alone [13]. The 2017 GOLD report recommended that therapy should be based on clinical criteria rather than isolated PFT [14]. Furthermore, due to the shortage of experienced spirometry experts in poor areas of developing countries, it’s hard to use PFT to screen asymptomatic patients in regular health examination on a large scale. Consequently, the need to develop new tools for early detection of COPD arises, and cost-effective strategies for case-finding are urgently needed.

In the past few years, growing evidences have shown that chest quantitative computer tomography (CT) has potential in COPD diagnosis and stratification [15–18]. CT-based imaging can help improve COPD detection and evaluation in patients who cannot undergo PFT [19]. In a recent study, deep learning (DL) models that utilize computed tomography (CT) image data were developed for automated detection and staging of spirometry-defined COPD. The result showed chest CT-DL approach could automatically identify spirometry-defined COPD and categorize patients according to the GOLD scale [11]. In the research reported by Lin Zhang, et al., they trained and tested the deep convolutional neural network (CNN) based on CT images of lung parenchyma and bronchial wall to determine the presence of COPD and GOLD staging, using PFT as reference, so as to infer lung function and determine the existence and severity of COPD. The result demonstrated that CNN can identify emphysema and airway wall remodeling on CT images to infer lung function and determine the existence and severity of COPD. As the CNN reached AUCs of 0.853 to determine the presence of COPD in the training and external test cohorts, and the accuracies of CNN to determine COPD GOLD grade in three- and five- classifications were 77.4 and 67.9%, respectively [20]. However, another recent study has shown that PFT results are not linearly correlated with CT lung attenuation areas in COPD patients [21], while the high radiation exposure is another important factor needed to be taken into account [22, 23]. The radiation exposure associated with CT has limited its use for COPD detection or frequent follow-up examinations to monitor disease progression [24]. Chest X-ray (CXR) is usually the first diagnostic tool used in evaluating patient’s lungs. Pulmonary emphysema is the main component of COPD characterized by permanent dilation of air spaces distal to terminal bronchioles [25]. Conventional CXR is commonly used to demonstrate the presence of emphysema in patients with suspected COPD [26–28]. It’s highly accurate for advanced emphysema [29], but only moderately sensitive in patients with mild to moderate emphysema [30–32]. Recently, machine learning (ML) technology is being assessed to perform medical tasks in almost every field of practice [33]. It has been successfully used in automated interpretation of PFT for differential diagnosis of obstructive lung diseases and COPD detection based on HRCT images [34–36]. However, none of the previous studies have used deep learning (DL) to predict COPD based on CXR images.

In the present study, we used DL algorithms to detect COPD and predict disease severity based on CXR imaging and clinical parameters, with the purpose to screen potential COPD patients while minimizing the need for additional radiographic examination. We hypothesized that applying DL algorithms to clinical and CXR imaging features would improve early diagnosis and prognosis prediction in COPD.

## Methods

### Study population

This was a multicenter retrospective study performed at the Third Affiliated Hospital of Sun Yat-sen University, the Third Affiliated Hospital of Sun Yat-sen University. Lingnan Hospital, the Six Affiliated Hospital of Guangzhou Medical University, Qingyuan People’s Hospital, and Huazhou Hospital of Traditional Chinese Medical. The data included frontal CXR images and clinical information of 1055 participants (535 patients with COPD and 520 controls) from outpatient, inpatient, and physical examination center settings between January 2019 and December 2021. This study was reviewed and approved by the Ethics Committee of the Third Affiliated Hospital of Sun Yat-sen University and requirements for written informed consent were waived due to the retrospective nature of the research.

Frontal images were identified by searching image databases for CXRs of the patients who also received PFT within one week, whereas lateral radiographs and oblique views were excluded. COPD diagnosis was confirmed by forced expiratory volume in 1 s (FEV1) to forced vital capacity (FVC) ratio less than 0.7 after inhalation of bronchodilators according to GOLD 2018. The severity of COPD is graded as GOLD 1 (FEV1%pred ≥ 80%), GOLD 2 (50%≤FEV1%pred < 80%), GOLD 3 (30%≤FEV1%pred < 50%), and GOLD 4 (FEV1%pred < 30%), as depending on the FEV1%pred value of PFT based on GOLD 2018. Since the diagnosis and severity of COPD depend on GOLD level based on PFT result, this study used the GOLD level as a reference standard to classify patients.

**Fig. 1:**
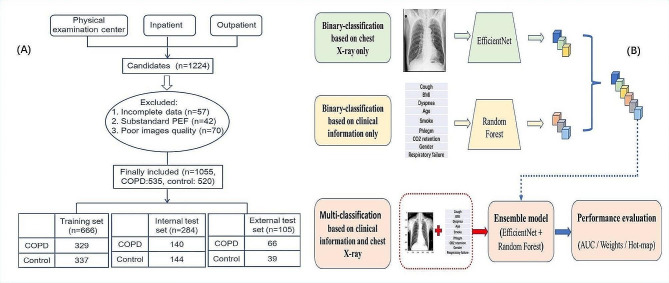
Diagram of the study procedure. (**A**) The inclusion and exclusion flow chart of the dataset; (**B**) Flowchart for proposed COPD detection and staging prediction model. Deep learning models were trained to classify COPD based on CXR images or clinical features only. The best two models were selected and mixed as a new model for simultaneously extracting clinical data and CXR images to identify COPD. COPD, chronic obstructive pulmonary disease; GOLD, the global initiative for chronic obstructive lung disease; PEF, pulmonary function test; CXR, chest X-ray; BMI, body mass index; AUC, area under the curve

As COPD usually occurs in patients older than 40 years [6], all the subjects included in the present study were > 40 years old. The exclusion criteria were: (1) pregnant women; (2) other pulmonary diseases with abnormal CXR presentations, such as bronchiectasis, pulmonary fibrosis, atelectasis, pulmonary infectious disease, active pulmonary tuberculosis, pleural effusion, lung cancer, and pneumothorax; (3) severe renal insufficiency, severe liver disease, human immunodefciency virus, or other immune-related diseases; (4) previous chest surgery; (5) severe cardiac insufficiency; (6) in the acute phase of COPD. A total of 1224 subjects ranging in age from 41 to 86 years were recruited. Spirometry data, demographic information, smoking history, clinical indices and manifestations information were collected using a standardized data collection form. After excluding cases with incomplete clinical data (57 cases), substandard pulmonary function (42 cases), and poor CXR image quality (70 cases), finally 1055 participants were enrolled in the cohort. The 950 subjects recruited from the Third Affiliated Hospital of Sun Yat-sen University and the Third Affiliated Hospital of Sun Yat-sen University. Lingnan Hospital were randomly split into training set and internal test set, with a ratio of 70%:30% (Fig. 1). For the training set (*n* = 666), 49.4% were COPD patients (*n* = 329). Of the 284 subjects in internal test set, 49.3% (*n* = 140) were COPD patients. The 105 participants (66 COPD patients and 39 control subjects) from the Six Affiliated Hospital of Guangzhou Medical University, Qingyuan People’s Hospital, and Huazhou Hospital of Traditional Chinese Medical were used for external test set.

### Demographic and clinical characteristics

A total of 1055 participants were finally included in the study: 535 COPD patients and 520 control subjects. The median age of COPD patients was higher than that of non-COPD participants (67 vs. 63, *P* < 0.001) and the majority of the COPD cohort was male (87.85%), which was consistent with COPD gender distributions in China [6]. A higher proportion of smokers (78.50% vs. 20.96%, *P* < 0.001), a reduced FEV1% (55.02% vs. 92.33%, *P* < 0.001), and a lower BMI (22.10 vs. 24.13, *P* = 0.001) were evident among COPD patients compared to control group. In addition, the symptoms of cough, sputum, and dyspnea were more common in COPD patients than in control subjects (77.94%, 51.40%, 50.47% vs. 13.46%, 8.46%, 2.31%, respectively). The percentages of stage 1, 2, 3, and 4 spirometry-defined COPD subjects on the GOLD scale were 18.50%, 42.06%, 25.61%, and 13.83%, respectively. Detailed demographic and clinical characteristics for the participants were provided in Table 1.

**Table 1:** Demographic and clinical characteristics

Demographic characteristics	Control (*n* = 520)	COPD (*n* = 535)	P value
Sex, %male (n)	55.19 (287)	87.85 (470)	*P* < 0.01
Age, *M* (IQR)	63 (41–85)	67 (45–86)	*P* < 0.01
BMI, mean (SD)	24.13 ± 3.29	22.10 ± 3.32	*P* = 0.01
Smoking, % (n)	20.96 (109)	78.50 (420)	*P* < 0.01
Cough, % (n)	13.46 (70)	77.94 (417)	*P* < 0.01
Sputum, % (n)	8.46 (44)	51.40 (275)	*P* < 0.01
Dyspnea, % (n)	2.31 (12)	50.47 (270)	*P* < 0.01
C0_2_ retention, % (n)	0.96 (5)	14.21 (76)	*P* < 0.01
Respiratory failure, % (n)	(0)	7.85 (42)	*P* < 0.01
FEV1% predicted, mean (SD)	92.33 ± 17.69	55.02 ± 19.82	*P* < 0.01
FEV1/FVC	85.17 ± 7.17	57.24 ± 11.31	*P* < 0.01
GOLD stage, % (n)			
1	NA	18.50 (99)	NA
2	NA	42.06 (225)	NA
3	NA	25.61 (137)	NA
4	NA	13.83 (74)	NA

*Abbreviations* COPD, chronic obstructive pulmonary disease; BMI, body mass index; *M* (IQR), median, interquartile range; SD, standard deviation; NA, not applicable; CO_2_: carbon dioxide; FEV1, forced expiratory volume in 1 s; FVC, force vital capacity; GOLD, the global initiative for chronic obstructive lung disease

### Data preprocessing

The data set consisted of clinical information and CXR images. The clinical information contained nine characteristics in all: gender, age, average body mass index (BMI), history of smoking, cough, expectoration, carbon dioxide retention, (arterial partial pressure of carbon dioxide greater than 45mmHg), dyspnea, respiratory failure (arterial partial oxygen pressure less than 60mmHg, with or without elevated carbon dioxide levels) and so on. Identity information including patient’s name, hospitalization ID and other DICOM information were removed from CXR images. In two classifications, the data were divided into normal control subjects without COPD (*n* = 520) and patients with COPD (*n* = 535). In three-classifications, the data were divided into three groups, normal (*n* = 520), GOLD 1/2 (*n* = 318), and GOLD 3/4 (*n* = 217), while in five-classifications, the data were divided into five groups, including normal (*n* = 520), GOLD 1 (*n* = 99), GOLD 2 (*n* = 225), GOLD 3 (*n* = 137), and GOLD 4 (*n* = 74).

### Development of the COPD detection and COPD staging model

We first studied the comparison between normal control subjects and patients with COPD based on clinical data only. Clinical information were preprocessed through sk-learn’s data preprocessing interface StandardScaler, by filling in the missing values and standardizing the data to (-1, 1) interval. Three classical machine learning algorithms, decision tree, support vector machine and random forest were selected for modeling. The random forest algorithm showing best area under the curve (AUC) was determined to be the best algorithm for feature mining and extraction of clinical inform clinical information. For classification of CXR images, we generally chose transfer learning for modeling. Three classical deep learning convolution neural networks such as EfficientNet-B5, ResNet50 and DenseNet were selected for modeling [37–39]. The EfficientNet-B5 algorithm showing best AUC was determined to be the best algorithm for feature extraction of CXR images.

As referred to a previous study [40], we next input the clinical information into the random forest model and synchronously input CXRs input the EfficientNe-B5 model to get the corresponding prediction probability values of the two modal data. Finally, the two prediction probability values were averaged to get the final prediction value. This kind of model which combined the features of the clinical information model with the features of the image model was called Ensemble model. Based on the Ensemble model algorithm for simultaneous extraction of text and images, we were able to make more comprehensive use of data and diagnose more accurately. Finally, the Ensemble model was used to model three-classification and five-classification problems as well (as shown in Fig. 1).

### Statistical analysis

Measurement data with normal distribution were presented as mean ± standard deviation (SD), which can reflect the overall trend and degree of variation, while data with non-normal distribution were presented as the median (M) and upper and lower quartile spacing (IQR), not affected by extreme values. Categorical variables were presented as numbers (%). The Wilcoxon signed-rank or Kruskal-Wallis tests were used for numerical variables, because they are non-parametric tests and suitable for quantitative data with non-normal distribution and do not need to satisfy normality. Fisher exact tests were used for categorical variables, and it is sensitive to small sample sizes. Statistical analysis was performed using the IBM SPSS statistics 20.0 software (SPSS). For two-way classification, the threshold of CNN-derived confidence was determined by the maximum value of AUC. The confidence threshold is determined by AUC maximization, which can achieve the best precision and recall ratio balance. The diagnostic performance of CNN was evaluated by AUC, accuracy, sensitivity, specificity, and F1 score. F1 score is the harmonic average of model accuracy and recall in machine learning. These indexes can be used to evaluate the application value of CNN model.

## Results

### Determining the presence of COPD

Of the three machine learning algorithms used to classify COPD based on clinical information only, the random forest algorithm showed best performance for detecting COPD in the internal test set, with an AUC of 0.963, while the sensitivity, specificity, NPV, PPV, and F1 score were 0.940, 0.880, 0.940, 0.890 and 0.910, respectively. The Random Forest improves generalization ability through ensemble learning and utilizes randomness and multiple decision trees to increase robustness against noise, making it more suitable for clinical information classification problems with limited samples and complex distributions, thus outperforming SVM and a single decision tree. When using deep learning to predict COPD only based on CXR images, the EfficientNet-B5 algorithm exhibited relatively robust performance, with an AUC of 0.946. The EfficientNet achieves the best balance among accuracy, parameter amount, and computational cost through automated neural architecture search, efficient network module design, extensive data augmentation and optimization techniques, making it outperform ResNet and DenseNet on image classification tasks.

**Fig. 2:**
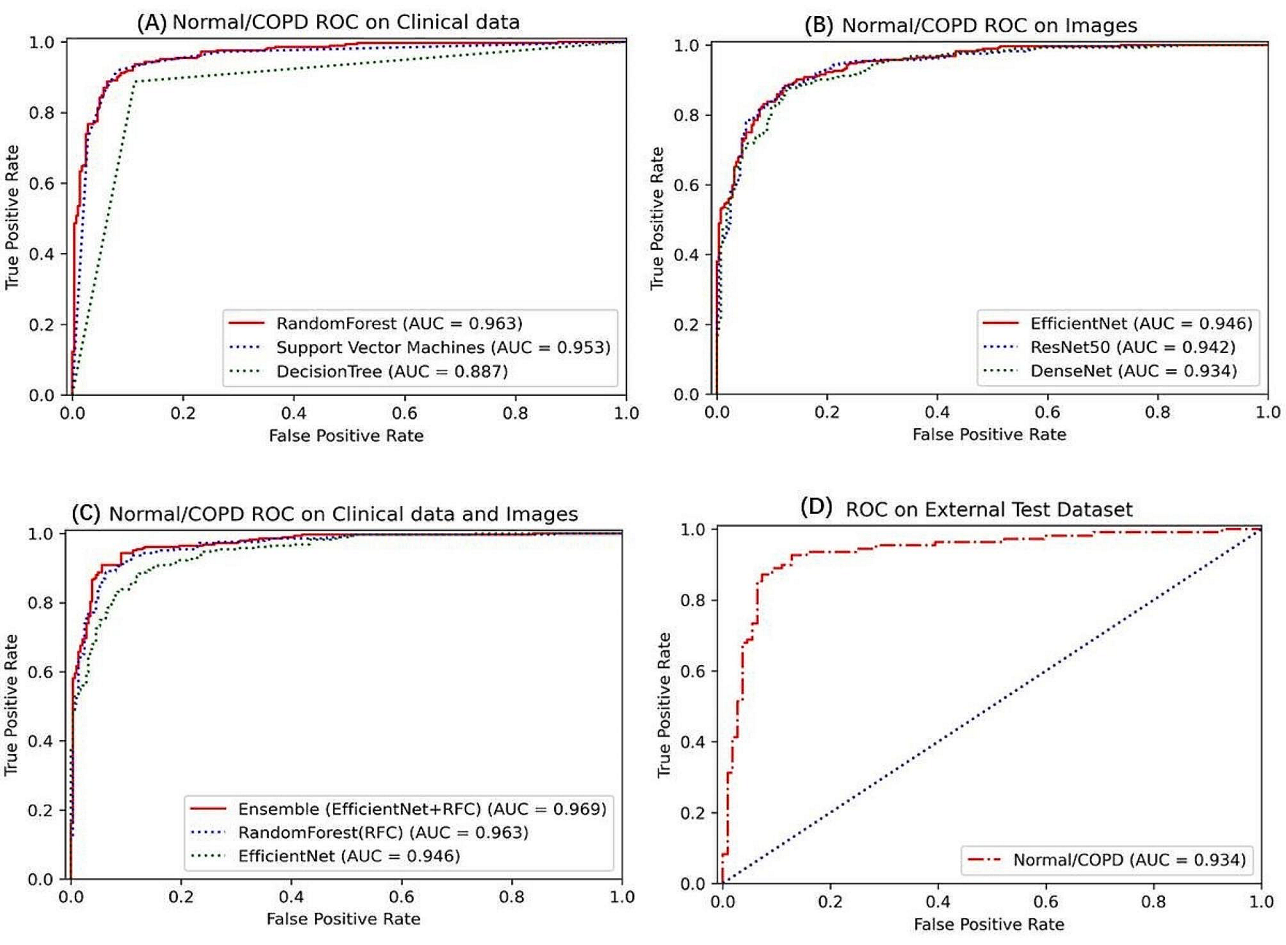
COPD detection performance of deep Learning models in two-classifications. AUC of COPD prediction based on clinical data (**A**) and CXR images (**B**) respectively in internal test; AUC for detecting COPD based on clinical data and CXR images simultaneously in internal test (**C**); AUC of COPD prediction based on clinical data and CXR images by Ensemble model in external test (**D**). AUC, area under the curve; COPD, chronic obstructive pulmonary disease; GOLD, the global initiative for chronic obstructive lung disease; CXR, chest X-ray

We subsequently applied Ensemble model to distinguish COPD by simultaneously extracting fusion features of clinical parameters and CXR images. The model showed an AUC of 0.969, slightly higher than better than that used clinical parameters or images only. We further incorporated Friedman’s statistical test and post hoc multiple comparisons into the analysis for more accurate comparison between these differences referring to the methods used in the previous studies [41, 42]. Friedman test was performed using the scipy library, resulting in a Friedman Statistic of 9.851 and a corresponding p-value of 0.00725, indicating significant differences among the groups. Subsequently, post hoc multiple comparisons were conducted using the scikit-posthocs library, specifically employing the Nemenyi test to discern specific group differences. The significance level between random forest and Ensemble is relatively high, suggesting that the differences between them are not highly significant. However, the significance levels between EfficientNet and Ensemble, as well as between EfficientNet and random forest, are relatively low, indicating significant differences between these pairs. It indicated that the Ensemble model simultaneously extracting fusion features of clinical parameters and CXR images could make more comprehensive use of data and diagnose more accurately (Fig. 2). Other measurements, including sensitivity, specificity, PPV, NPV, and F1 score were 0.960, 0.860, 0.870, 0.960 and 0.920 respectively, as summarized in Table 2.

**Table 2:** Prediction performance of DL models for COPD based on CXR images and clinical data in internal test

	Based on CXR images only				Based on clinical data only				Multi-mode		
		EfficientNet	ResNet50	DenseNet		Random Forest	Support Vector	Decision Tree		Ensemble	
AUC		0.946	0.942	0.934		0.963	0.953	0.887		0.969	
ACC		0.890	0.880	0.880		0.910	0.910	0.890		0.920	
PPV		0.860	0.860	0.840		0.890	0.880	0.860		0.870	
NPV		0.910	0.890	0.920		0.940	0.950	0.920		0.960	
Sensitivity		0.910	0.890	0.920		0.940	0.960	0.920		0.960	
Specificity		0.860	0.860	0.830		0.880	0.860	0.850		0.860	
F1		0.890	0.880	0.880		0.910	0.920	0.890		0.920	

*Abbreviations* DL, deep learning; CXR, chest x ray; COPD, chronic obstructive pulmonary disease; AUC, area under the curve; ACC, accuracy; PPV, positive predictive value; NPV, negative predictive value; F1, false positive rate

External test was further performed with the Ensemble model to verify the generalization ability of the learning algorithms in diagnosing COPD. It showed an AUC of 0.934 in COPD prediction based on the external test set, just slightly declined compared to that in internal test (Fig. 2).

### Prediction performance of COPD staging

**Fig. 3:**
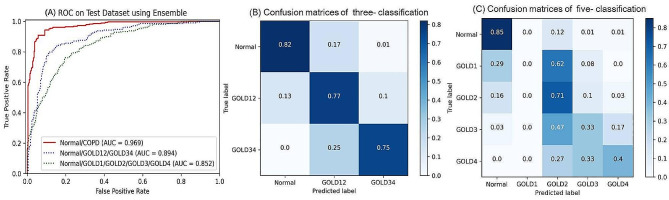
GOLD stage prediction performance of the Ensemble model based on CXR images and clinical data. (**A**). AUC of COPD stage prediction for the Ensemble model in two, three and five-classifications. Confusion matrices of the Ensemble model to evaluate COPD stage in three (**B**) and five-classifications (**C**). GOLD, the global initiative for chronic obstructive lung disease; COPD, chronic obstructive pulmonary disease; AUC, area under the curve

We next used the Ensemble model to evaluate disease severity of COPD by predicting different GOLD stages according to clinical data and CXR images. For three-classification, the AUC reached 0.894 and the accuracy was 0.79 (shown in Fig. 3). In five-classification model, the AUC value slightly declined to 0.852, with an accuracy of 0.52 (eTable 1).

### Interpretability of clinical information and feature extraction visualization

**Fig. 4:**
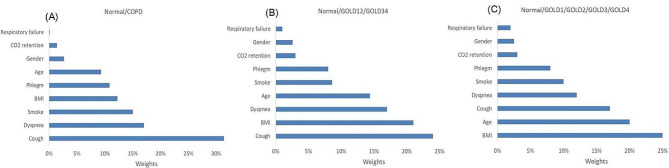
Weight distribution maps for interpretability of clinical information in COPD detection (**A**), stage prediction in three-classifications (**B**), and five-classifications (**C**) respectively. COPD, chronic obstructive pulmonary disease; GOLD, the global initiative for chronic obstructive lung disease; BMI, body mass index

Through the feature importance interface of random forest, we could rank the importance of 9 kinds of clinical information to model decision-making in ascending order. For two classifications, three classifications and five classifications, different weight distribution maps were shown in Fig. 4 respectively.

Gradient-weighted class activation mapping (Grad-CAM) was applied to visualize feature extraction by using a heatmap, the main signature lesions related to COPD detection in CXR images, such as emphysema was manifest as increased values in the GradCAM results (eFigure 1 in the supplement).

## Discussion

In this multicenter retrospective study, we used deep learning algorithms to predict COPD based on clinical parameters and CXR images, with the purpose to find a more sensitive and simple option for early detection of potential COPD patients. The diagnostic accuracies of COPD reached an AUC of 0.965 in internal test and 0.934 in external test to detect COPD according clinical characteristics and CXR images, better than the results of previous study, which used deep residual networks for automated detection of COPD based on low dosed CT images only, with an ACU of 0.889 [4] and 0.899 [20], respectively. The DL models also retained high accuracies in determining COPD grade in three-and five-classifications, showed an AUC of 0.894 and 0.79 respectively. It indicated that the algorithms had considerable potential to screen suspected COPD patients, which would help early diagnosis of the disease and subsequently increase the rate of smoking cessation and preventive treatment, so as to improve prognosis of COPD.

As we know, the diagnosis of COPD mainly depends on PFT. However, its accuracy is highly dependent on the patient’s cooperation, which explains the common under and over diagnosis of COPD in clinical practice [35, 43]. To address this challenge, recently some other methods have been considered to be useful in early screening of COPD as rapid advance on ML technology [44]. Chest CT has also been widely used to detect lung texture abnormalities and assess the state of COPD [45, 46]. In a recent prospective study, the pulmonary ventilation function of COPD was assessed by analyzing chest CT images, with an accuracy of 88% and an AUC value of 0.82 [47]. In another study, DL models that utilize CT image for detection and staging of COPD achieved an AUC of 0.934 on the internal test set and 0.866 on the external test [11]. These studies indicated better performance of DL based on CT images. However, proper selection and capture of target images of a large amount of image data is still a question to be resolved even with the help of ML, while high radiation exposure may also limit the use of CT for early screening of COPD.

To develop a more simple and effective method for COPD detection, we focused on conventional CXR imaging. This method is economical and safe but rarely been used for COPD detection due to insensitivity, which, however, can be overcome with the help of DL. As expected, the AUC achieved 0.946 on detecting COPD, approximated to the AUC of 0.934 that achieved by DL models used CT images and clinical information [11], and better that the result of another study published by Tang LYW, which used deep residual networks for automated detection of COPD based on low dosed CT images only, with an ACU of 0.889 [7]. It indicated that the DL model based on CXR images was able to perform well in COPD screening. According to the guideline of GOLD, diagnosis of COPD should be made based on symptoms meanwhile. The clinical parameters, such as symptoms and smoking history, are equally important in diagnosis of COPD [12, 48]. Thus, to increase the sensitivity of models, clinical parameters including demographic data, symptoms and examination results were combined with CXR images for assessment in this study. Consequently, the AUC rise to 0.969, higher than that used clinical parameters (0.963) or CXR images (0.946) only. What’s more, the model kept good performance even in external test (with an AUC of 0.943). This might help our algorithm better generalize to detection of COPD in patients without significant airflow limitations.

The weight distribution maps showed that different parameters accounted different importance in decision-making. Of which, type 2 respiratory failure and CO_2_ retention ranked in the bottom as the least two important factors in predicting COPD. This was not in consistence with the situation in clinical practice, as the two situation above were mostly seen in sever COPD patients. The possible explanation might be that the COPD patients included in the present study were mainly mild to moderate, who rarely had CO_2_ retention or type II respiratory failure. Only 11.51% patients had CO_2_ retention and 5.76% showed type II respiratory failure. As a result, it seemed like that the two parameters above accounted not so important in decision-making of predicting COPD consequently. However, when the same model was used to evaluate disease severity, the weight distribution of them increased in three- and five- classifications as they were quite common manifestation for sever patients of GOLD stage 3 or 4.

Cough and dyspnea are most common symptoms of COPD, and smoking is the most important cause. They also played critical roles in discriminating COPD patients from control subjects, and ranked the top three on the decision-making weight distribution map, just as respected. Nevertheless, when the Ensemble model was applied to predict disease stages, e.g., in five-classifications, the two leading parameters in decision-making changed to BMI and age, suggesting that they contributed more important in assessing disease severity. As we know, BMI and age are closely correlated to pulmonary function. As the age advances, the pulmonary function gets worse, while BMI is usually positively correlated to pulmonary function. Weight loss and decreased BMI seem to be more common in severer COPD patients such as patients with GOLD stage 3 or 4.

One of the common problems seen in DL algorithm is the “black box” nature of the DL model, which may greatly limit its use in clinical situations, as it does not provide sufficient information for clinicians concerning its decision-making process. Yet the lack of transparency in machine learning can be overcome by applying gradient-weighted class activation mapping (Grad-CAM) to visualize feature extraction using a heatmap [49]. Through the gradcam interface of the EfficientNet model, we can get the hot focus areas of the CNN model for the diagnosis of COPD patients. The results indicated that the model paid specific attention to these lesions when distinguishing COPD subjects. This could make doctors and patients better understand what they have learned from DL, and whether they can rest assured that ML can assist them in diagnosis.

There are several limitations in the present study. First, this was a retrospective study only performed in four medical institutes. The outer generalization of the deep learning algorithms needed to be tested further by prospective study including more centers. Second, the prediction was performed based on COPD and normal control. Subjects involved in the present study were mainly COPD patients without other complications or disease, as radiographic images with other lesion were eliminated. For better use in clinical practice, subsequent study with larger sample size including complication or comorbidities of COPD is needed. Third, a limited number of subjects with GOLD 1 and 4 were enrolled, which might constrain the model’s stratification capacity and resulted in the discrepancy of staging efficiency between groups. To improve the efficiency of detection and staging, we are currently recruiting more participants and aim to optimize our cohort in the future.

In conclusion, we developed a more simple, sensitive and safer learning approach for detecting and staging of COPD. The proposed model approach achieved the desired performance and could serve as a powerful tool for COPD screening and evaluation, which may help clinicians easily identify possible suspected COPD patients. Nevertheless further studies are necessary to determine the feasibility of these outcomes in a prospective clinical setting.

### Electronic supplementary material

Below is the link to the electronic supplementary material.

Supplementary Material 1

## Data Availability

The datasets generated and analysed during the current study are not publicly available due for protection of participants’ privacy, but are available from the corresponding author on reasonable request.

## Abbreviations

COPD: Chronic obstructive pulmonary disease
PFT: pulmonary function test
GOLD: Global Initiative for Chronic Obstructive Lung Disease
CT: computed tomography
CXR: chest X-ray
ML: machine learning
DL: deep learning
FEV1: forced expiratory volume in 1 s
FVC: forced vital capacity
BMI: body mass index
SD: standard deviation
AUC: area under the curve
CO2: carbon dioxide
ACC: accuracy
PPV: positive predictive value
NPV: negative predictive value
F1: false positive rate
Grad-CAM: Gradient-weighted class activation mapping
