# Supplementary material for: Screening and staging of chronic obstructive pulmonary disease with deep learning based on chest X-ray images and clinical parameters
Source: BMC Pulm Med. 2024 Mar 26;24:153. doi: 10.1186/s12890-024-02945-7 (PMC10964626; doi:10.1186/s12890-024-02945-7)
Supplement: Supplementary file 1 — Supplementary Material 1 [file 12890_2024_2945_MOESM1_ESM.docx]

**eTable 1.** GOLD stage prediction performance of the Ensemble model.

**eFigure 1.** Feature extraction visualization using Heatmaps of Grad-CAM for COPD detection based on CXR images. The chest x-ray image of COPD patient (A); The Grad-CAM of the same COPD patient (B).

**eTable 1. GOLD Stage Prediction Performance of the Ensemble Model**

| Classification | AUC | ACC | sensitivity | F1 |
| --- | --- | --- | --- | --- |
| Three classification | 0.894 | 0.79 | 0.78 | 0.78 |
| Five classification | 0.852 | 0.52 | 0.59 | 0.55 |

Abbreviations: GOLD, the global initiative for chronic obstructive lung disease; AUC, area under the curve; ACC, accuracy; F1, false positive rate.


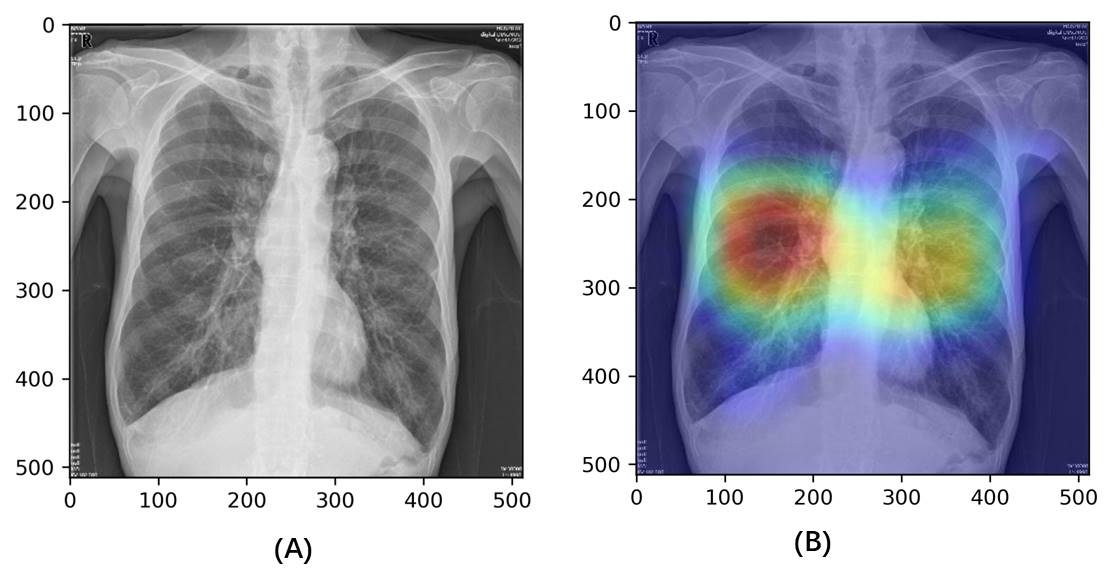


eFigure 1. Feature extraction visualization using Heatmaps of Grad-CAM for COPD detection based on CXR images. The highlighted areas with increased values are discriminative features for identification of COPD. The chest x-ray image of COPD patient (A); The Grad-CAM of the same COPD patient (B). COPD, chronic obstructive pulmonary disease; Grad-CAM, Gradient Class Activation Map.
